# Language Variations in Describing Nutrition and Hydration Interventions in State Physician Orders for Life Sustaining Treatment Forms and the Implications for Advanced Dementia Patients

**DOI:** 10.1089/pmr.2023.0029

**Published:** 2024-01-05

**Authors:** Sarah Rebecca Stephen

**Affiliations:** Center for Medical Humanities, Compassionate Care, and Bioethics, Renaissance School of Medicine, Stony Brook University, Stony Brook, New York, USA.

**Keywords:** advance directives, bioethics, dementia, geriatric palliative care end of life issues, long-term care nursing home patients at the end of life

## Abstract

**Background:**

Research suggests that language can impact medical decision-making, but few studies exist describing the variations in language to describe end-of-life nutrition and hydration interventions. The language contained in the Physician Orders for Life Sustaining Treatment (POLST) form varies across states, but this variation has not yet been fully analyzed. This investigation has implications for communicating with surrogates about the insertion of feeding tubes in advanced dementia patients, a common procedure in this population despite its potentially high risks and low benefits.

**Objective:**

Identify and analyze the variations in language related to end-of-life nutrition and hydration interventions in state POLST forms.

**Design:**

Descriptive study.

**Measurements:**

The most up-to-date POLST forms for each of the 50 US states and the District of Columbia as of August 2022 were analyzed for their descriptions of end-of-life nutrition and hydration interventions.

**Results:**

Fifty out of 51 (98%) forms referenced nutrition and/or hydration interventions. Four main modifiers of the word “nutrition” and/or “hydration” were identified: artificial (32%), artificially administered (56%), medically administered (14%), and assisted/medically assisted (18%). Forty-eight (96%) forms indicated an explicit option to forgo feeding tubes, and all of these forms described doing so with negatively valenced language.

**Conclusions:**

The language describing end-of-life nutrition and hydration interventions and feeding tubes in state POLST forms is insufficiently specific and varies significantly across the country. These terms are at best ambiguous and at worst imply incorrect information. More precise language may assist in the difficult discussion between physicians and surrogates about inserting feeding tubes in advanced dementia patients.

## Introduction

At the heart of effective medical care is informed consent. Patients or their surrogates must be given the information necessary to understand medical procedures. The American Medical Association states that informed consent should include discussions of the purpose, risks, and benefits of a given medical treatment, including forgoing that treatment.^1^ However, if this discussion contains opaque, misleading, or insufficiently specific language—even if this inaccuracy or imprecision is not the intention of the medical practitioner—then informed consent is not established.

The precision of language is particularly important when describing end-of-life nutrition and hydration interventions in advanced directives. Engaging with these documents can be an emotionally charged experience for families, and the procedures that patients or surrogates select are critical. Thus, it is valuable to examine the language in these documents and analyze how it may impact medical decision-making. Physician Orders for Life-Sustaining Treatment (POLST), also known as Medical Orders for Life-Sustaining Treatment, is the main form that outlines patient preferences for end-of-life treatment. Each state as well as the District of Columbia has its own variation of the POLST, which may include directives for cardiopulmonary resuscitation, antibiotic usage, and feeding preferences, among other medical interventions. However, there is no uniform language used to describe these various procedures. In particular, there are significant differences in language from state to state regarding end-of-life nutrition and hydration. Analyzing these variations may help the medical community better understand how to improve communication with patients and their surrogates, thus better establishing informed consent.

Several studies have suggested that differences in language can impact medical decision-making.^2–4^ However, to our knowledge, no study has examined the variation in language used to describe end-of-life nutrition and hydration interventions, especially for advanced dementia patients. This subject is notable because the discussion around tube feeding for this specific population, especially percutaneous endoscopic gastronomy (PEG) tubes, is contentious.^5^ PEG tubes were introduced in the early 1980s as an alternative to hand feeding in nursing homes.^6^ Although studies have found that PEG tubes for advanced dementia patients do not improve the quality or length of life, substantial numbers of elderly patients still receive permanent feeding tubes every year.^5,7–13^ Approximately one-third of advanced dementia patients have feeding tubes in place.^14^

The usage of a tube to transmit nutrition into the body can take other forms besides PEG tubes. These include nasojejunal (NJ) tubes, nasogastric (NG) tubes, and jejunostomy (J) tubes. In contrast with PEG tubes, which are permanent placements, these other types of tubes are often temporary measures. Often, however, all of these interventions are often described with the blanket term “feeding tube.” Thus, examining the language used to describe PEG tubes and other kinds of feeding tubes may shed light on how to better inform patients and surrogates of the risks and benefits of these medical interventions.

## Methods

The POLST form or an equivalent document for each of the 50 United States and the District of Columbia (“the states”) were identified through the national POLST website at www.polst.org. If not located on this website, the state POLST document was found via an Internet search or through state-specific public health and POLST websites. Document collection occurred in June, July, and August of 2022. All 51 POLST forms were located and the most up-to-date version available was used for analysis.

Each document was examined for any mention of nutrition and/or hydration interventions. All states' POLST documents except for Arkansas' contained explicit mention of such interventions. Each mention of nutrition and hydration in the remaining 50 forms was cataloged. These phrasings were linguistically analyzed by considering their semantic connotations and potential implications. The wording to describe refusing a feeding tube was also cataloged and flagged for negatives such as “no/not” or for word choice with a negative valence such as “avoid.”

## Results

### Feeding tubes

Forty-one states (84%) had POLST forms with explicit use of the term “tube” or feeding tube (Table 1). The word “tube” was used in three different phrases: “surgically placed tubes” (10%), “feeding tubes” (34%), and “nutrition by tube” (40%). A few forms also used the medical terminology of “total enteral nutrition” (MS, OK), “total parenteral nutrition” (MS, OK, CT), or in one case as simply the abbreviation “PEG” (AL).

**Table 1.** Usage of “Tube” Across State Physician Orders for Life Sustaining Treatment Documents

Wording using “tube”	States	% of state POLST forms with phrase
“New or existing surgically-placed tubes”	AL, AK, AZ, NH, WV	10%
“Feeding tube(s)”	CA, ID, IL, KS, KY, MO, MS, NE, NY, NC, MS, OK, RI, SC, TX, UT, VA	34%
“Nutrition by tube”	CO, DC, FL, GA, HI, IN, IA, OH, LA, ME, MN, MT, ND, OR, PA, SC, SD, TN, WI, WY	40%

POLST, Physician Orders for Life Sustaining Treatment.

### Nutrition and hydration modifiers

Across all 50 POLST forms, four main modifiers of the words “nutrition” and “hydration” were identified: “artificial” (32%), “artificially administered” (56%), “medically administered” (14%), and “medically assisted” (18%) (Table 2). These terms are not sufficiently specific and may engender confusion. Furthermore, many states group nutrition and hydration into a single category. Critically, nutrition and hydration are not equivalent actions. Long-term feeding tubes may impose more serious burdens and complications than fluids. Lack of fluid may cause death much more swiftly than lack of nutrition, and administering fluids imposes less of a burden than administering nutrition.^7^ However, these two distinct interventions are often grouped when making medical decisions.

**Table 2.** Modifiers of Nutrition and Hydration Across State Physician Orders for Life-Sustaining Treatment Documents

Modifier of nutrition and/or hydration	States	% of state POLST forms with phrase
Artificial	AL, AK, AZ, CA, CO, DE, FL, GA, HI, IN, IA, ID, LA, ME, MA, MN, MT, ND, NH, NJ, NM, OR, PA, RI, SC, TN, VT, WV, WI, WY, UT, WA (32%)	32%
Artificially administered	CA, CO, DE, FL, GA, HI, IN, IA, LA, ME, MD, MN, MT, ND, NE, NJ, NM, NY, OH, OR, PA, SC, SD, TN, VA, VT, WI, WY (56%)	56%
Medically assisted	AL, AK, AZ, DC, MI, NH, TX, WA, WV (18%)	18%
Assisted	OK (2%)	2%
Medically administered	CT, IL, KS, KY, MS, MO, NC (14%)	14%

#### Artificial nutrition and hydration

There is ambiguity in the usage of the term “artificial.” “Artificial” may indicate that the nutrition and/or hydration are artificial in ingredients and quality. Alternatively, “artificial” may suggest that the method by which the nutrition and/or hydration is taken in is artificial. The usage of “artificial” as a modifier for nutrition specifically also makes one think of its antonyms like “natural” or “real.” With the concept of “natural” or “real” food being akin to healthy food, there may be a sense that “artificial” nutrition is inferior. “Artificial” nutrition also calls to mind chemical preservatives or additives, so the health content of this medical intervention may be questioned. Practically speaking, the content of the nutrition of a feeding tube should meet the nutrition needs of the patient. This means a balanced blend of nutrients, which may come from pre-made formulas composed of blended whole foods or prepared blends of whole foods made in a blender at home.

#### Artificially administered nutrition

“Artificially administered” conveys that the nutrition enters the body through a vehicle deemed artificial rather than through using one's own hands or mouth to take in food directly. Thus, it is the usage of a vessel to transport the food and liquids from the outside world into the body of the patient that makes the intervention artificial. By this logic then, spoons, forks, thermoses, and especially straws are similarly artificial in the way that a tube may be seen as artificial. None are part of the human body and all are inventions to facilitate the consumption of food. In fact, as testified at the 1988 trial concerning the case of Nancy Cruzan, Dr. Ronald Cranford considered even spoon-feeding to be under the umbrella of “artificial” as it did not enter the body through a “natural route”.^15^ However, there is nothing necessarily more artificial about the nutrition contained in a smoothie eaten by mouth via a straw rather than placed into a feeding tube.

#### Medically administered nutrition and hydration

“Medically administered” may indicate that nutrition is administered in a medical setting, which may not always be the case. The feeding tube is inserted in a medical setting, but the delivery of the nutrition is then usually done at home or a skilled nursing facility. Furthermore, “medically” may insinuate that the process is not a medical procedure to insert a tube but instead that the nutrition will be administered by medical personnel, such as a physician, nurse, or another health care worker, perhaps as hand feeding. “Medically” could also refer to the setting in which the nutrition and hydration are administered, such as a hospital. Thus, the permanence of this intervention may not be made clear. Feeding tubes are usually permanently placed in advanced dementia patients, not temporarily used only in a hospital setting. Alternatively, a benefit of the term “medically” may indicate that the insertion of a tube is a medical procedure, which makes it clearer that a feeding tube is not a necessity but simply one of many optional medical interventions. Thus, in the case of advanced dementia patients, the term “medically administered” may convey that inserting a feeding tube is not necessarily required or preferable.

#### Assisted/medically assisted nutrition and hydration

The usage of “medically assisted,” similarly to “medically administered,” also may indicate that nutrition is administered in a medical setting rather than as a medical procedure. The term “to assist” implies that a task is shared by at least two entities. The usage of “assisted” rather than “administered” may also wrongly imply that the patient has some ability to feed themselves and only requires minor help from a medical professional, which is generally not the case. Thus, this term potentially ascribes more agency to the patient than appropriate, especially for patients who have advanced dementia. Notably, the word “administered” is more impersonal than “assist”; “assist” is an altruistic action, while “administered” has a more detached connotation.

#### Forgoing feeding tubes

The language around forgoing feeding tubes is overwhelmingly negative in connotation. Forty-eight (96%) POLST forms indicated a specific option to forgo feeding tubes, and all of these forms did so using negatively valenced language (Table 3). No document had an option for an alternative phrase such as “palliative nutrition” or “comfort feeding.” Terms with negation or a negative valence are more likely to be seen as undesirable options.^2^ This is, for example, one of the reasons that terms such as “allow natural death” and “comfort care” are increasingly becoming more popular than the term “do not resuscitate.” It is important to underscore that not inserting a feeding tube is not synonymous with starving a patient. A person nearing death will often naturally stop eating.^16^ Patients are provided with fluids and small pieces of easy-to-consume food, such as applesauce or ice chips, according to their needs.

**Table 3.** Negatively Valenced Words to Describe Forgoing Feeding Tubes Across State Physician Orders for Life-Sustaining Treatment Documents

Modifier of nutrition and/or hydration	States	% of state POLST forms with phrase
“No artificial means of nutrition”	CA	2%
“No medically administered nutrition”	CT	2%
“Feeding tube” (with Yes/No checkboxes)	ID	2%
“Do not provide artificially administered nutrition”	MD	2%
“No nutrition by tube”	OH	2%
“No tube feeding”	OK	2%
“Do not insert feeding tube”	SC	2%
“Preference is to avoid medically assisted nutrition”	WA	2%
“withhold or stop treatment” of several listed procedures, including “medically assisted provisions of nutrition”	MI	2%
“No medically assisted nutrition”	TX	2%
“Do not administer artificial nutrition”	VT	2%
“No medically assisted nutrition by tube”	DC	2%
“No parenteral nutrition”	MS, OK	4%
“No medically administered means of nutrition, including feeding tubes”	IL, KS, MO	6%
“No artificial nutrition”	DE, IN, MA, NJ, NM, RI, UT	7%
“No artificial means of nutrition desired”	AL, AK, AZ, NH, WV	10%
“No feeding tube”	KY, MS, NE, NY, NC, VA	12%
“No artificial nutrition by tube”	CO, FL, GA, HI, IA, LA, ME, MN, MT, ND, OR, PA, TN, WI, WY	30%

## Discussion

There is significant variation in how state POLST forms refer to nutrition and hydration. There need to be discussions about standardizing how medical practitioners communicate with patients and surrogates about their options. That the majority of state POLST documents used general terms like “feeding tube” or “nutrition by tube” underscores this tendency toward generic, nonspecific wording instead of precise descriptions when discussing this treatment. “Tube” and “feeding tube” are not sufficiently specific. Additionally, the four linguistic modifiers of nutrition and hydration may at best be ambiguous and at worst imply incorrect information. Couching the decision in vague terminology such as “medically assisted” or “artificially administered” does nothing more than obscure important medical information. Furthermore, neutral language for opting out of feeding tubes is needed. The ideal terms should make it clear that feeding tubes are medical procedures, and further information should be required to explain the procedure and its outcomes on POLST forms. Explicitly writing “feeding tube” with clarification of whether this is a PEG, NG, NJ, or J-tube or an intravenous line, and explanations of what these options mean medically will give patients and surrogates appropriate information and terminology to better understand these interventions. More specific language may assist in the difficult discussion between physicians and surrogates about inserting feeding tubes in advanced dementia populations.

There is also a need for neutral language options to discuss forgoing the insertion of a feeding tube. Each state POLST form used a term with a negative valence to describe declining the insertion of a feeding tube. Rather than have patients or surrogates check a box that says “Does not want a feeding tube,” or a variation as shown in Table 3, a palliative option that connotes a sense of humanity and dignity may be appropriate. Suggested terms to indicate the refusal of tube insertion for an advanced dementia patient could include “comfort feeding” and “palliative nutrition and hydration.” Although wordier, “allow natural appetite to dictate feeding and hydration,” “allow natural appetite and thirst cessation,” or “feed according to appetite and thirst” may also be appropriate.

## Future Research

This article characterized the language of nutrition and hydration interventions in state POLST documents. Further investigation is needed to identify other terminology and phrasings for nutritional interventions and feeding tubes beyond those in these forms. Research is also needed to characterize patient and clinician perceptions of these terms and to determine whether their usage affects medical decision-making. Ultimately, this research could result in more specific and standardized language regarding end-of-life nutrition and hydration interventions.

## Abbreviations Used

J: jejunostomy
NG: nasogastric
NJ: nasojejunal
PEG: percutaneous endoscopic gastronomy
POLST: Physician Orders for Life Sustaining Treatment
